# 4-(2,3-Dimethyl­phen­yl)piperazin-1-ium chloride monohydrate

**DOI:** 10.1107/S1600536808019016

**Published:** 2008-06-28

**Authors:** Imen Ben Gharbia, Riadh Kefi, Meher El Glaoui, Erwann Jeanneau, Cherif Ben Nasr

**Affiliations:** a Laboratoire de Chimie des Matériaux, Faculté des Sciences de Bizerte, 7021 Zarzouna, Tunisia; b Universtié Lyon1, Centre de Diffractométrie Henri Longchambon, 43 boulevard du 11 Novembre 1918, 69622 Villeurbanne Cedex, France

## Abstract

The title compound, C_12_H_19_N_2_^+^·Cl^−^·H_2_O, contains a network of 4-(2,3-dimethyl­phen­yl)piperazin-1-ium cations, water mol­ecules and chloride anions. The crystal packing is influenced by O—H⋯Cl, N—H⋯Cl, N—H⋯O, C—H⋯O and C—H⋯Cl hydrogen bonds, resulting in structure with an open-framework architecture.

## Related literature

For related literature, see: Ben Gharbia *et al.* (2005 ▶, 2007 ▶); Bernstein *et al.* (1995 ▶); Pajewski *et al.* (2004 ▶); Sessler *et al.* (2003 ▶); Schmidtchen & Berge (1997 ▶). For the refinement weighting scheme, see: Prince (1982 ▶); Watkin (1994 ▶).
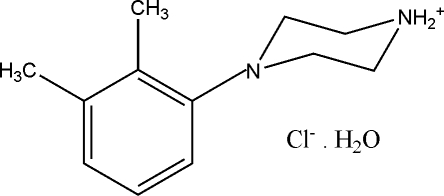


## Experimental

### 

#### Crystal data

C_12_H_19_N_2_^+^·Cl^−^·H_2_O
*M* *_r_* = 244.76
Triclinic, 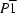

*a* = 7.5439 (3) Å
*b* = 9.4204 (3) Å
*c* = 10.4347 (4) Å
α = 72.733 (2)°
β = 74.152 (2)°
γ = 70.250 (2)°
*V* = 654.05 (4) Å^3^
*Z* = 2
Mo *K*α radiation
μ = 0.28 mm^−1^
*T* = 150 K
0.13 × 0.12 × 0.09 mm

#### Data collection

Nonius KappaCCD diffractometer
Absorption correction: none
5719 measured reflections
3073 independent reflections
2601 reflections with *I* > 2σ(*I*)
*R*_int_ = 0.016

#### Refinement

*R*[*F* ^2^ > 2σ(*F* ^2^)] = 0.036
*wR*(*F* ^2^) = 0.035
*S* = 1.10
2491 reflections
145 parameters
H-atom parameters constrained
Δρ_max_ = 0.25 e Å^−3^
Δρ_min_ = −0.20 e Å^−3^

### 

Data collection: *COLLECT* (Nonius, 2001 ▶); cell refinement: *DENZO*/*SCALEPACK* (Otwinowski & Minor, 1997 ▶); data reduction: *DENZO*/*SCALEPACK*; program(s) used to solve structure: *SIR97* (Altomare *et al.*, 1999 ▶); program(s) used to refine structure: *CRYSTALS* (Betteridge *et al.*, 2003 ▶); molecular graphics: *DIAMOND* (Brandenburg, 1998 ▶); software used to prepare material for publication: *CRYSTALS*.

## Supplementary Material

Crystal structure: contains datablocks global, I. DOI: 10.1107/S1600536808019016/cf2207sup1.cif

Structure factors: contains datablocks I. DOI: 10.1107/S1600536808019016/cf2207Isup2.hkl

Additional supplementary materials:  crystallographic information; 3D view; checkCIF report

## Figures and Tables

**Table 1:** Hydrogen-bond geometry (Å, °)

*D*—H⋯*A*	*D*—H	H⋯*A*	*D*⋯*A*	*D*—H⋯*A*
N2—H3⋯Cl1	0.90	2.18	3.069 (1)	169
N2—H4⋯O1^i^	0.91	1.86	2.776 (2)	175
O1—H1⋯Cl1	0.82	2.32	3.120 (1)	165
O1—H2⋯Cl1^ii^	0.83	2.31	3.136 (1)	171
C10—H15⋯Cl1^iii^	0.99	2.87	3.846 (1)	168
C12—H20⋯Cl1^iv^	0.97	2.84	3.779 (3)	161
C12—H19⋯O1^v^	0.99	2.73	3.448 (2)	130

Symmetry codes: (i) 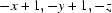; (ii) 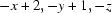; (iii) 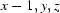; (iv) 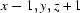; (v) 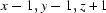.

**Fig. 1.**
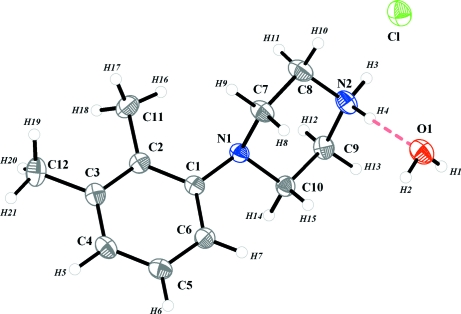
The asymmetric unit of (I), showing 40% probability displacement ellipsoids.

**Fig. 2.**
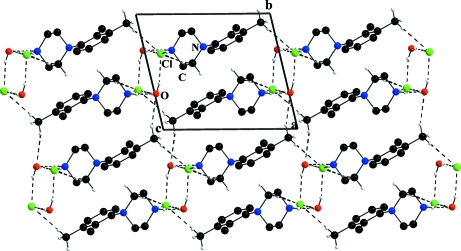
The packing of (I), viewed down the a axis, showing the O—H···Cl, N—H···Cl, N—H···O, C—H···O and C—H···Cl interactions (dashed lines) between the 4-(2,3-dimethylphenyl)piperazin-1-ium cation, water molecule and chloride anion.

**Fig. 3.**
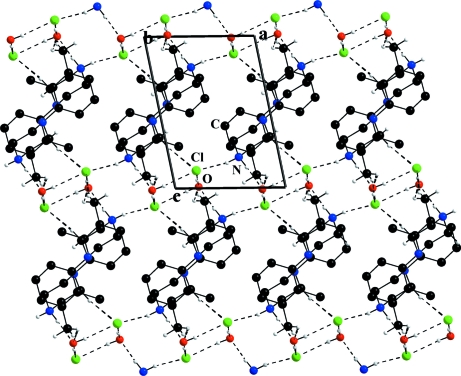
The packing of (I), viewed down the b axis, showing the zigzag character of the structure. Hydrogen bonds are indicated by dashed lines.

## supplementary crystallographic information

### Comment

The coordination chemistry of anions is a fast-growing area of supramolecular chemistry (Schmidtchen & Berge, 1997), on account of the importance of anion binding, recognition and transport in many biochemical processes (Pajewski *et al.*, 2004). Thus, the Cl^-^ anion has been successfully used to assemble double-helical motifs of various molecules (Sessler *et al.*, 2003). Here a new member of this family, the title compound, is presented, which was obtained during our studies of the preparation of new organic hydrochloride compounds. As shown in Fig. 1, the asymmetric unit of the crystal structure of the title compound contains a 4-(2,3-dimethylphenyl)piperazin-1-ium cation, a chloride anion and a water molecule, associated in a hydrogen-bonded network. Two water molecules and two Cl^-^ anions are interconnected through O—H···Cl hydrogen bonds, forming an 8-membered ring with graph-set *R*_2_^4^(8) Bernstein *et al.*, 1995). These entities are connected to two antiparallel organic cations *via* N—H···Cl, N—H···O and C—H···Cl hydrogen-bonding interactions to construct a convoluted hydrogen-bonded chain which runs in the c-axis direction (Fig. 2). When projected along the *b* axis, the chains have a marked zigzag structure and somewhat resemble a helix (Fig. 3). In addition to the hydrogen-bonding associations to Cl1 and O1, the organic cations have a second role by linking these chains to each other to form layers parallel to the bc plane through C—H···O hydrogen bonds. Fig. 3 shows that these planes are interconnected by NH_2_^+^ groups to form an open framework architecture through hydrogen-bond interactions. An examination of the organic group geometrical features shows that the carbon atoms in the benzene ring of the title compound have a good coplanarity and they form a conjugated ring with an average deviation of 0.013 Å. The mean value of the C—C bond lengths [1.3967 (17) Å], which is between a single bond and a double bond, agrees with that in phenylpiperazinium tetrachloridozincate(II) [1.384 (4) Å] (Ben Gharbia *et al.*, 2005). The piperazine-1,4-diium ring of the title compound adopts a typical chair conformation and its geometric parameters [d_av_(C—N) = 1.4818 (16) and d_av_(C—C) = 1.5437 (17) Å] are in full agreement with those found in 4-(2,3-dimethylphenyl)piperazin-1-ium tetrachloridozincate(II) (Ben Gharbia *et al.*, 2007).

### Experimental

An aqueous 1*M* HCl solution and 1-(2,3-dimethylphenyl)piperazine in a 1:1 molar ratio were mixed and dissolved in sufficient ethanol. Crystals of (I) grew as the ethanol evaporated at 293 K over the course of a few days.

### Refinement

The H atoms were all located in a difference map, but those attached to carbon atoms were repositioned geometrically. The H atoms were initially refined with soft restraints on the bond lengths and angles to regularize their geometry (C—H in the range 0.93–0.98, N—H in the range 0.86–0.89 and O—H = 0.82 Å) and *U*_iso_(H) (in the range 1.2–1.5 times *U*_eq_ of the parent atom), after which the positions were refined with riding constraints. Low-angle reflections possibly affected by the beam-stop and some other outliers were omitted from the refinement.

### Crystal data

**Table:**

C_12_H_19_N_2_^+^·Cl^–^·H_2_O	*Z* = 2
*M**_r_* = 244.76	*F*_000_ = 264
Triclinic, *P*1	*D*_x_ = 1.243 Mg m^−^^3^
Hall symbol: -P 1	Mo *K*α radiation λ = 0.71069 Å
*a* = 7.5439 (3) Å	Cell parameters from 2750 reflections
*b* = 9.4204 (3) Å	θ = 0.4–27.9º
*c* = 10.4347 (4) Å	µ = 0.28 mm^−^^1^
α = 72.733 (2)º	*T* = 150 K
β = 74.152 (2)º	Block, colorless
γ = 70.250 (2)º	0.13 × 0.12 × 0.09 mm
*V* = 654.05 (4) Å^3^	

### Data collection

**Table:**

Nonius KappaCCD diffractometer	2601 reflections with *I* > 2σ(*I*)
Monochromator: graphite	*R*_int_ = 0.016
*T* = 150 K	θ_max_ = 27.9º
φ and ω scans	θ_min_ = 2.1º
Absorption correction: none	*h* = −9→9
5719 measured reflections	*k* = −12→12
3073 independent reflections	*l* = −13→13

### Refinement

**Table:**

Refinement on *F*	Hydrogen site location: inferred from neighbouring sites
Least-squares matrix: full	H-atom parameters constrained
*R*[*F*^2^ > 2σ(*F*^2^)] = 0.036	*w* = [1-(*F*_o_-*F*_c_)^2^/36σ^2^(*F*)]^2^/[0.443T_0_(x) + 0.129T_1_(x) + 0.131T_2_(x)] where T_i_ are Chebychev polynomials and x = *F*_c_/*F*_max_ (*P*rince, 1982; Watkin, 1994)
*wR*(*F*^2^) = 0.035	(Δ/σ)_max_ = 0.0004
*S* = 1.10	Δρ_max_ = 0.25 e Å^−^^3^
2491 reflections	Δρ_min_ = −0.20 e Å^−^^3^
145 parameters	Extinction correction: None
Primary atom site location: structure-invariant direct methods	

### Fractional atomic coordinates and isotropic or equivalent isotropic displacement parameters (Å^2^)

**Table:**

	*x*	*y*	*z*	*U*_iso_*/*U*_eq_	
C1	−0.09326 (17)	0.26100 (14)	0.53871 (13)	0.0211	
C2	−0.08069 (18)	0.18226 (14)	0.67481 (13)	0.0224	
C3	−0.24822 (19)	0.15986 (14)	0.76843 (13)	0.0243	
C4	−0.42381 (18)	0.21709 (15)	0.72522 (14)	0.0270	
C5	−0.43454 (18)	0.29399 (16)	0.59077 (14)	0.0285	
C6	−0.26999 (18)	0.31526 (15)	0.49687 (13)	0.0255	
C7	0.18840 (18)	0.14963 (15)	0.38467 (14)	0.0276	
C8	0.38588 (19)	0.16564 (16)	0.30991 (15)	0.0313	
C9	0.24578 (19)	0.45044 (15)	0.26085 (13)	0.0260	
C10	0.05184 (17)	0.42554 (14)	0.33543 (13)	0.0230	
C11	0.1080 (2)	0.12337 (17)	0.72288 (15)	0.0320	
C12	−0.2398 (2)	0.07656 (18)	0.91485 (14)	0.0353	
Cl1	0.77820 (5)	0.34324 (4)	0.11653 (4)	0.0340	
O1	0.78625 (14)	0.68790 (12)	0.00994 (10)	0.0353	
N1	0.07922 (14)	0.28419 (12)	0.44468 (11)	0.0216	
N2	0.36750 (15)	0.31276 (13)	0.20308 (12)	0.0278	
H1	0.7848	0.5995	0.0526	0.0528*	
H2	0.9007	0.6873	−0.0182	0.0528*	
H3	0.4845	0.3270	0.1661	0.0430*	
H4	0.3144	0.3085	0.1358	0.0437*	
H5	−0.5385	0.2058	0.7916	0.0322*	
H6	−0.5565	0.3352	0.5610	0.0341*	
H7	−0.2770	0.3659	0.4045	0.0295*	
H8	0.1198	0.1410	0.3200	0.0321*	
H9	0.2040	0.0561	0.4575	0.0315*	
H10	0.4588	0.0790	0.2644	0.0369*	
H11	0.4582	0.1695	0.3749	0.0356*	
H12	0.3114	0.4642	0.3220	0.0314*	
H13	0.2299	0.5429	0.1850	0.0309*	
H14	−0.0266	0.5162	0.3773	0.0269*	
H15	−0.0141	0.4180	0.2684	0.0273*	
H16	0.2087	0.1594	0.6514	0.0467*	
H17	0.1484	0.0098	0.7464	0.0473*	
H18	0.0940	0.1606	0.8047	0.0484*	
H19	−0.1476	−0.0282	0.9197	0.0537*	
H20	−0.2011	0.1364	0.9587	0.0526*	
H21	−0.3671	0.0679	0.9639	0.0532*	

### Atomic displacement parameters (Å^2^)

**Table:**

	*U*^11^	*U*^22^	*U*^33^	*U*^12^	*U*^13^	*U*^23^
C1	0.0204 (5)	0.0193 (5)	0.0239 (6)	−0.0065 (4)	−0.0025 (4)	−0.0057 (5)
C2	0.0250 (6)	0.0184 (5)	0.0251 (6)	−0.0079 (5)	−0.0054 (5)	−0.0040 (5)
C3	0.0289 (6)	0.0211 (6)	0.0243 (6)	−0.0104 (5)	−0.0022 (5)	−0.0059 (5)
C4	0.0243 (6)	0.0269 (6)	0.0298 (7)	−0.0099 (5)	0.0020 (5)	−0.0098 (5)
C5	0.0213 (6)	0.0308 (7)	0.0331 (7)	−0.0073 (5)	−0.0039 (5)	−0.0081 (6)
C6	0.0231 (6)	0.0273 (6)	0.0250 (6)	−0.0079 (5)	−0.0040 (5)	−0.0040 (5)
C7	0.0241 (6)	0.0215 (6)	0.0347 (7)	−0.0077 (5)	0.0025 (5)	−0.0088 (5)
C8	0.0229 (6)	0.0274 (7)	0.0392 (8)	−0.0073 (5)	0.0025 (5)	−0.0088 (6)
C9	0.0289 (6)	0.0260 (6)	0.0255 (6)	−0.0128 (5)	−0.0024 (5)	−0.0061 (5)
C10	0.0236 (6)	0.0224 (6)	0.0220 (6)	−0.0081 (5)	−0.0026 (5)	−0.0034 (5)
C11	0.0286 (7)	0.0337 (7)	0.0317 (7)	−0.0115 (6)	−0.0113 (5)	0.0038 (6)
C12	0.0452 (8)	0.0378 (8)	0.0246 (7)	−0.0210 (7)	−0.0034 (6)	−0.0016 (6)
Cl1	0.02788 (17)	0.0430 (2)	0.03519 (18)	−0.01831 (14)	−0.00224 (13)	−0.00861 (14)
O1	0.0303 (5)	0.0377 (6)	0.0367 (5)	−0.0128 (4)	0.0007 (4)	−0.0094 (4)
N1	0.0205 (5)	0.0191 (5)	0.0234 (5)	−0.0059 (4)	−0.0013 (4)	−0.0044 (4)
N2	0.0229 (5)	0.0332 (6)	0.0291 (6)	−0.0136 (4)	0.0028 (4)	−0.0101 (5)

### Geometric parameters (Å, °)

**Table:**

C9—C10	1.5176 (17)	C6—H7	0.947
C9—N2	1.4986 (17)	C5—C4	1.3851 (19)
C9—H12	0.966	C5—H6	0.968
C9—H13	0.985	C4—C3	1.3957 (19)
C10—N1	1.4686 (16)	C4—H5	0.965
C10—H14	1.005	C3—C2	1.4070 (17)
C10—H15	0.993	C3—C12	1.5038 (19)
C7—C8	1.5159 (17)	C2—C11	1.5090 (17)
C7—N1	1.4701 (16)	C12—H21	0.975
C7—H9	0.974	C12—H20	0.974
C7—H8	0.991	C12—H19	0.993
C8—N2	1.4900 (18)	C11—H18	0.983
C8—H11	0.995	C11—H16	0.981
C8—H10	0.989	C11—H17	0.981
C1—C6	1.3979 (17)	O1—H1	0.822
C1—C2	1.4060 (17)	O1—H2	0.831
C1—N1	1.4391 (15)	N2—H3	0.900
C6—C5	1.3886 (18)	N2—H4	0.914
			
C10—C9—N2	109.56 (10)	C4—C5—H6	120.8
C10—C9—H12	111.0	C5—C4—C3	120.72 (12)
N2—C9—H12	108.8	C5—C4—H5	120.5
C10—C9—H13	110.3	C3—C4—H5	118.7
N2—C9—H13	108.5	C4—C3—C2	119.63 (12)
H12—C9—H13	108.6	C4—C3—C12	119.84 (12)
C9—C10—N1	109.41 (10)	C2—C3—C12	120.53 (12)
C9—C10—H14	109.0	C3—C2—C1	119.20 (11)
N1—C10—H14	108.9	C3—C2—C11	119.40 (12)
C9—C10—H15	108.6	C1—C2—C11	121.39 (11)
N1—C10—H15	111.3	C3—C12—H21	109.5
H14—C10—H15	109.6	C3—C12—H20	109.5
C8—C7—N1	110.04 (10)	H21—C12—H20	107.6
C8—C7—H9	108.4	C3—C12—H19	110.4
N1—C7—H9	109.2	H21—C12—H19	109.8
C8—C7—H8	109.4	H20—C12—H19	109.9
N1—C7—H8	110.2	C2—C11—H18	109.7
H9—C7—H8	109.5	C2—C11—H16	110.8
C7—C8—N2	109.88 (11)	H18—C11—H16	109.0
C7—C8—H11	110.1	C2—C11—H17	110.4
N2—C8—H11	107.8	H18—C11—H17	108.5
C7—C8—H10	111.2	H16—C11—H17	108.4
N2—C8—H10	108.0	H1—O1—H2	107.1
H11—C8—H10	109.9	C7—N1—C10	109.62 (10)
C6—C1—C2	120.31 (11)	C7—N1—C1	112.16 (9)
C6—C1—N1	121.24 (11)	C10—N1—C1	115.19 (10)
C2—C1—N1	118.45 (11)	C9—N2—C8	112.04 (10)
C1—C6—C5	119.86 (12)	C9—N2—H3	107.5
C1—C6—H7	119.9	C8—N2—H3	109.3
C5—C6—H7	120.2	C9—N2—H4	108.6
C6—C5—C4	120.27 (12)	C8—N2—H4	110.2
C6—C5—H6	118.9	H3—N2—H4	109.1

### Hydrogen-bond geometry (Å, °)

**Table:**

*D*—H···*A*	*D*—H	H···*A*	*D*···*A*	*D*—H···*A*
N2—H3···Cl1	0.90	2.18	3.069 (1)	169
N2—H4···O1^i^	0.91	1.86	2.776 (2)	175
O1—H1···Cl1	0.82	2.32	3.120 (1)	165
O1—H2···Cl1^ii^	0.83	2.31	3.136 (1)	171
C10—H15···Cl1^iii^	0.99	2.87	3.846 (1)	168
C12—H20···Cl1^iv^	0.97	2.84	3.779 (3)	161
C12—H19···O1^v^	0.99	2.73	3.448 (2)	130

Symmetry codes: (i) −*x*+1, −*y*+1, −*z*; (ii) −*x*+2, −*y*+1, −*z*; (iii) *x*−1, *y*, *z*; (iv) *x*−1, *y*, *z*+1; (v) *x*−1, *y*−1, *z*+1.
